# Leprosy: A Review of Epidemiology, Clinical Diagnosis, and Management

**DOI:** 10.1155/2022/8652062

**Published:** 2022-07-04

**Authors:** Kou-Huang Chen, Cheng-Yao Lin, Shih-Bin Su, Kow-Tong Chen

**Affiliations:** ^1^School of Mechanical and Electronic Engineering, Sanming University, Sanming, Fujian, China; ^2^Division of Hematology-Oncology, Department of Internal Medicine, Chi-Mei Medical Center, Liouying, Tainan, Taiwan; ^3^Department of Senior Welfare and Services, Southern Taiwan University of Science and Technology, Tainan, Taiwan; ^4^Department of Environmental and Occupational Health, National Cheng Kung University, Tainan, Taiwan; ^5^Department of Occupational Medicine, Chi-Mei Medical Center, Tainan, Taiwan; ^6^Department of Occupational Medicine, Tainan Municipal Hospital (Managed By Show Chwan Medical Care Corporation), Tainan, Taiwan; ^7^Department of Public Health, College of Medicine, National Cheng Kung University, Tainan, Taiwan

## Abstract

Leprosy is a neglected infectious disease caused by acid-fast bacillus *Mycobacterium leprae*. It primarily affects the skin and then progresses to a secondary stage, causing peripheral neuropathy with potential long-term disability along with stigma. Leprosy patients account for a significant proportion of the global disease burden. Previous efforts to improve diagnostic and therapeutic techniques have focused on leprosy in adults, whereas childhood leprosy has been relatively neglected. This review aims to update the diagnostic and therapeutic recommendations for adult and childhood leprosy. This review summarizes the clinical, bacteriological, and immunological approaches used in the diagnosis of leprosy. As strategies for the diagnosis and management of leprosy continue to develop better and more advanced knowledge, control and prevention of leprosy are crucial.

## 1. Introduction

Leprosy, or Hansen's disease, is a chronic bacterial infection caused by *Mycobacterium leprae* (*M. leprae*) infection [1]. *M. leprae*, the taxonomic order Actinomycetales, family Mycobacteriaceae, is an acid-fast, gram-positive obligate intracellular bacillus that demonstrates tropism for phagocytes in the skin and Schwann cells within peripheral nerves [2]. Although the 9-banded armadillo infects the wild in the southern United States, *M leprae* grows in the footpads of mice, which is the main method of growing *M leprae* in laboratories around the world [3].

**Figure 1:**
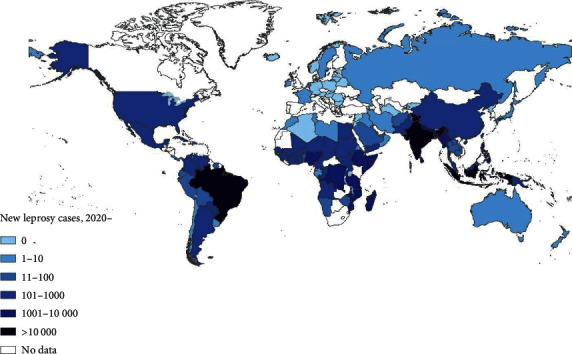
Global distribution of new leprosy cases in 2020 (source: World Health Organization/National Leprosy Program, WHO 2021) [6].

Leprosy is ubiquitous in tropical countries, particularly underdeveloped and developing countries. In 1990, the World Health Organization (WHO) proposed the global goal of eliminating leprosy by the end of the 20th century [4]. Despite the commitment of governments, researchers, and healthcare workers worldwide, disease control has not yet been achieved. Between 1900 and 2000, although the number of new leprosy cases remained relatively constant or slightly increased owing to intensified case-finding efforts, a significant reduction in the number of registered cases for treatment and prevalence of cases was observed during this period because of the effectiveness of multidrug therapy (MDT) and improvement in the quality of health care in patients with leprosy worldwide [5].  Figure 1 shows the geographical distribution of new leprosy cases worldwide in 2020 [6]. This indicates that the highest rates for the detection of new cases are reported by countries in the African region (AFR) and Southeast Asia region (SEAR). Of the 127 countries that reported in 2020, India, Brazil, and Indonesia continued to report the highest number of new cases (>10,000); of the 124 countries that provided data on child cases, SEAR accounted for 62% of all new-child cases. In 2016, the WHO launched a new global strategy entitled “The Global Leprosy Strategy 2016–2020: Accelerating toward a leprosy-free world” with the main objectives of reducing the number of children diagnosed with leprosy and presenting visible physical deformities to zero, all countries enacting specific legislation against discrimination, and the reduction of new leprosy cases with grade 2 disability to less than one case per million [7].

Despite control efforts, including the widespread use of MDT and stabilization of reported new case detection rates in the last few years, leprosy remains endemic in many developing countries [8, 9]. The number of patients undergoing treatment at the end of 2019 was 202,256, with a corresponding prevalence rate of 22.9 per million people, of which 14,893 were children below 14 years of age [4]. In particular, cases still appear in various countries in Southeast Asia, America, Africa, the Eastern Pacific, and the Western Mediterranean [4, 9]. Moreover, the high incidence rate in children under 15 years of age is important, indicating that there is early exposure of the population to the bacillus, which is associated with an elevated prevalence in the general population and is a good indicator of high transmission and poor quality of control programs [10–16]. In the absence of an effective vaccine, early diagnosis and treatment of the disease are important to stop the transmission of *M. leprae,* reduce the risk of physical disability and deformity, and reduce the physical, psychosocial, and economic burden of the disease [17–19]. Due to difficulties in diagnosis, lack of scientific studies on leprosy, and largely unknown outcomes in patients with leprosy, childhood leprosy reflects early exposure to *M. leprae* but remains neglected. Therefore, this review aimed to describe the recent advances in the epidemiology, clinical diagnosis, and management of leprosy.

## 2. Classification

**Figure 2:**
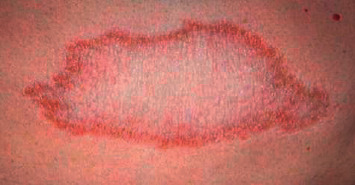
Tuberculoid leprosy: lesion with a single, stable, hairless plaque, and well-defined borders (photograph courtesy of Eichelmann, et al.) [23].

**Figure 3:**
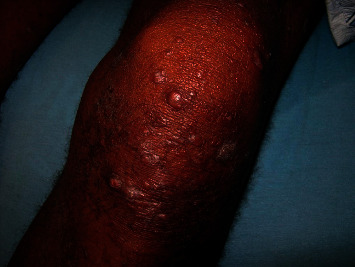
Lepromatous leprosy: lesion with diffuse thickening, numerous discrete, and confluent nodules (photograph courtesy of White, et al.) [24].

Table 1 presents a comparison of the proposed leprosy classification. Leprosy was first classified by Rabello, and the characteristics of disease polarity have been established [4, 20]. In 1966, Ridley–Jopling introduced a classification method for clinical leprosy based on the patient's clinical characteristics and immune status [21]. According to this classification system, the disease is divided into two poles and an intermediate state, including polar tuberculoid leprosy (TT) (Figure 2), borderline tuberculoid leprosy (BT), mid-borderline leprosy (BB), borderline lepromatous leprosy (BL), and lepromatous leprosy (LL) (Figure 3) [21–24].

Patients with a strong cell-mediated immune reaction had few lesions with low or undetectable mycobacteria and were classified as having tuberculoid forms, whereas patients anergic to *M. leprae* had multiple lesions with higher loads of mycobacteria and were classified as having lepromatous forms [21]. Where an affected person falls within the classification model depends on their immune response [22]. Tuberculoid forms show little evidence of *M. leprae-specific* antibodies but a vigorous T helper (Th)1 cytokine response, whereas lepromatous forms show a Th2 cytokine response with markedly high antibody titers but T-cell hypo-response (anergy) [19, 25]. The balance of the Th1/Th2 response alone cannot fully explain the response to leprosy. Other T-cell subsets have been identified to play an important role in determining host immunity [25]. Tuberculoid leprosy is stable, rarely contagious, or self-limiting. The bacillus is not detectable by bacteriological analysis, but the Mitsuda reaction (lepromin test) is positive, and granulomas are typically found on biopsy. Borderline cases are classified as borderline lepromatous, borderline tuberculoid, or mid-borderline leprosy according to the pole (lepromatous or tuberculoid) they tend toward [21, 22]. Patients who have not yet developed a cell-mediated immune response to organisms are classified as having indeterminate leprosy (IL) [26]. If left untreated, they can progress to either tuberculoid or lepromatous disease.

In 1982, the World Health Organization (WHO) established a simplified classification based on the bacterial index (BI) (density of leprosy bacilli in slit-skin examination) to access medical care in regions where medical resources are insufficient and divided the disease into paucibacillary (PB) and multibacillary (MB) cases. PB indicates those who have a BI lower than 2+, and MB patients have a BI higher than or equal to 2+ [27]. In 1988, the WHO Expert Committee on Leprosy recommended that treatment should be initiated prior to smear tests; thus, practical and rapid methods of classification were developed that do not require expensive diagnostic equipment and do not put first-line healthcare workers at risk [22]. According to this classification, PB cases are defined as those in which less than five skin lesions and/or only one nerve trunk is involved, whereas MB cases involve more than five skin lesions and/or more than one nerve trunk. However, this classification system is not perfect, because most MB cases are misclassified as PB cases with unsuitable treatment. The classification of leprosy patients into multibacillary and paucibacillary groups determines the duration of treatment. Misclassification leads to an increased risk of relapse due to insufficient treatment if a multibacillary patient is classified as having paucibacillary disease. This prolongs the time at which a patient is infected. There are reports in which the results of serological and bacteriological approaches have been found to agree substantially [28]. Slit-skin smears (SSS) with a demonstration of bacilli in biopsies (bacterial index of granuloma or BIG) are the most sensitive and effective method for identifying multibacillary cases [29]. Ridley's logarithmic scale or bacterial index was used to interpret the test results, which were recorded as a number followed by a plus mark to express the degree of abundance or scarcity of bacteria per field [23]. It has been suggested that the bacterial index of granulomas should be estimated during the diagnostic workup of paucibacillary patients.

All the above-mentioned classifications were also used to classify the clinical forms of childhood leprosy [20]. It is assumed that the largest number of childhood leprosy cases is in the indeterminate clinical form; however, there was an average proportion (23%) of this form of the disease in a study of the Brazilian childhood population [10]. A delayed diagnosis may be the reason for this paradox.

## 3. Leprosy Reactions

Leprosy reactions are caused by an immune response between the host and *M. leprae.* Leprosy reactions are an important consequence of permanent nerve damage during leprosy [30]. Leprosy reactions include acute/subacute inflammatory processes that mainly involve the skin and nerves and are the primary cause of morbidity and neurological disability. They may occur regularly at any stage of the disease, even without treatment [30]. However, this reaction can also be initiated or aggravated by effective chemotherapy due to the active destruction of bacilli during or after treatment, thereby producing an abundance of antigenic material in the immune system [31, 32]. Leprosy reactions can be subdivided into types 1 and 2.

**Figure 4:**
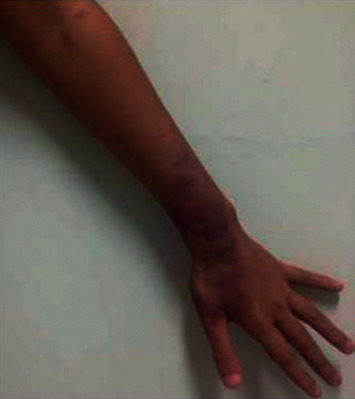
Type 1 reaction: lesions with erythema, swelling, papules, and plaques (photograph courtesy of Oliveira, et al.) [20].

Type 1 reactions (Figure 4) are type IV cell-mediated allergic hypersensitivity reactions that most commonly occur in the BT, BB, and BL forms [20, 33]. This was also described as a reversal reaction. The mechanisms of these reactions involve cellular immune responses against mycobacterial antigens [32, 33]. Type 1 reactions can be improved (reversal reactions) or worsened (degradation reactions). In these cases, the common clinical manifestations are hyperesthesia, erythema, edema, subsequent scaling, and sometimes ulceration and neuritis [32, 34]. Approximately 95% of type 1 reaction cases occur in the first 2 years after starting MDT [35]. Timely and effective treatment before irreversible damage occurs is important in patients with type 1 reactions.

**Figure 5:**
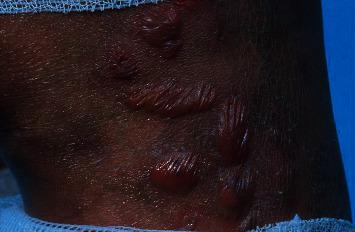
Type 2 reaction: lesions with erythema multiform-like bullous (photograph courtesy of Alemu Belachew, et al.) [36].

A type 2 reaction (Figure 5), erythema nodosum leprosum (ENL), is a type III humoral hypersensitivity reaction [36, 37]. The ENL reaction is immunologically characterized by immune complex deposition in the tissues, blood, and lymphatic vessels [38]. It usually occurs most frequently in patients with LL and occasionally in those with BL. ENL can occur at any time during the course of leprosy but usually occurs in the first year after MDT treatment [39]. The most common manifestation of type 2 reactions is the rapid appearance of painful erythematous subcutaneous nodules that may ulcerate [37]. Type 2 reactions are accompanied by systemic symptoms such as fever, with changes in the patient's general health status. Approximately 60% of patients with lepromatous leprosy develop type 2 reactions that may recur several times during the course [37]. Physical and mental stress, multiple drug therapy, vaccines, pregnancy, surgical procedures, injuries, intercurrent infections, and other antibacterial treatments are exacerbating factors [36].

Lucio's phenomenon is a clinical variant of ENL or a third rare type of reaction. It usually occurs in Central and South America and in immigrants from these areas, although cases have also been reported in Europe and Asia [40]. The major clinical manifestations are red congestive macules that progress to blisters, hemorrhagic infarcts, and necrotic sloughs. Eventually, irregular atrophic scars are left behind [38, 41]. Other findings include alopecia of the eyebrows and eyelashes, destructive rhinitis with nasal septal perforation, eruptive telangiectasia, hepatosplenomegaly, and lymphadenopathy [42]. If not treated immediately, patients can die owing to superinfection and sepsis [43].

Generally, children do not present with leprosy. Some studies have shown a low frequency of lepra reactions, varying between 1.36% and 29.7% [44–46]. Type 1 reactions were the most common, given that the most frequent clinical form is borderline BT [20]. Although children have a low risk of morbidity with *leprae* reactions, children with neural thickening have a higher risk of developing deformities (10–30%) than those without neural enlargement [47–49].

Taken together, leprosy reactions are consequences of the dynamic immune response to *M. leprae* that may occur before, during, or after the completion of MDT for leprosy. It is the main cause of nerve damage and disability in children with leprosy. Children are a vulnerable social group and cannot be expected to seek help from health professionals. Therefore, it is important to ensure that families are involved in monitoring the signs and symptoms of leprosy in their children.

## 4. Diagnosis

### 4.1. Clinical Findings

Clinical evaluation is the first step in the diagnosis of leprosy and is generally sufficient in most cases. However, one of the challenges in diagnosing leprosy is simply to consider this disease in the list of differential diagnoses, particularly in developed countries where leprosy has almost been eradicated or is extremely rare [26]. Obtaining travel or family history (e.g., adopted or immigrated from an endemic area) is important when considering a diagnosis of leprosy. In addition, practical information about the protective measures of the care team (e.g., high index of suspicion and wearing gloves) to prevent transmission should be included.

Skin lesions are usually the first clinical manifestation observed. If appropriate medical treatment is not received, leprosy may progress to cause permanent damage to the skin, nerves, limbs, and other organs [36]. WHO experts have listed the main diagnostic criteria as follows [22]: (1) a hypopigmented or erythematous skin lesion or reddish skin patch with definite loss of sensation; (2) a thickened or enlarged peripheral nerve with loss of sensation and/or weakness of muscle supplied by the nerve; and (3) a positive acid-fast skin smear or bacilli observed in a skin smear/biopsy. When all three signs were present, diagnostic accuracy was as high as 95% [26, 36].

The borderline TT form was the most common clinical type in children, followed by the borderline BT form [48, 50]. In children, a single lesion exists in the exposed area, and a small portion of the lesions are present (<8%) in the gluteal region [51–53]. In other words, the entire body is likely to present leprosy lesions. Therefore, nerve endings may be involved in the early stages of leprosy. In some patients, *M. leprae* invades both sensory and autonomic nerves, causing a reduction in cutaneous sensation and absence of sweating [19, 20]. However, musculoskeletal presentations also cause leprosy in the differential diagnosis of other autoimmune diseases such as juvenile idiopathic arthritis [54].

The clinical diagnosis of leprosy is dependent on the recognition of disease signs and symptoms and is therefore only possible once the disease has manifested. Physical examination does not identify the early stages of the disease when clinical manifestations are rare [36, 53]. Previous studies showed that untrained health practitioners may not be effective in recognizing early signs of the disease [36, 55]. It is likely that clinical diagnosis is delayed or even missed, especially in regions where leprosy is controlled [56]. This finding might be related to a long delay between disease onset and diagnosis as well as a high rate of disability in grade 2 among new-child patients. In addition, it is difficult to conduct a thermal reaction test in younger children, which is necessary for the differential diagnosis of other childhood cutaneous lesions. Simple and objective tests to detect leprosy infection are necessary to assist clinicians in the early diagnosis of leprosy and to detect leprosy before its clinical signs manifest.

### 4.2. Slit-Skin Smear Test

Bacilloscopic examination is an important method for accurate diagnosis. The preferred sites for sample collection are active lesions or lesions with altered sensitivity, as well as the ear lobes and contralateral elbow [57]. In the absence of injury, intradermal shaving can be performed in both the ear lobes and elbows [50]. The slit-skin smear test has a specificity of 100% and sensitivity of 50% [57–59]. A smear from the nasal mucosa, ear lobe, forehead, chin, extensor surfaces of the forearms, knee, cooler parts of the body, and/or skin lesions was the preferred site for sample collection. After collection, Fite staining or modified Ziehl–Neelsen staining was used to examine acid-fast bacilli (AFB) and calculate the Ridley logarithmic scale or bacterial index (BI) score [23, 60]. A positive result indicated that the patient had MB. However, a negative result does not rule out a clinical diagnosis of leprosy and does not necessarily classify the patient as having PB. The AFB staining technique requires the presence of at least 10^4^ organisms per gram of tissue for reliable detection under a microscope; thus, organisms have a very low sensitivity of detection [60].

Microscopic examination revealed positive bacilli (9.3%–25%) in children [12, 44]. Household contact is an important risk factor for infection in children [61]. According to Cuba's experience, 89% of diagnosed cases have at least one case of leprosy in their family [62]. Therefore, a family history can be used as a diagnostic tool.

### 4.3. Skin Biopsy and Histopathologic Examination

Skin biopsy is an important diagnostic tool for leprosy. A biopsy was obtained from the leading margins of the most recent and active skin lesions with the entire thickness of the dermis, at least a portion of the subcutaneous fat lesion, and stained according to the Fite-Faraco method [63, 64]. Tissue samples were used for the diagnosis. They were collected from lesions on the body, stained with hematoxylin-eosin and Fite tissue stains, and examined for the type, extent of involvement, infiltrate characteristics, and AFB. Biopsy specimens may be further analyzed for granuloma fraction, bacterial index of granuloma (BIG) for grading AFB in tissues, and histopathological index [23]. BIG is a method used to detect AFB bacilli in a given tissue volume [26, 29]. Histopathological examination can be useful for verifying the type of leprosy and differentiating a leprosy reaction [23, 64]. Histopathological findings are used as criteria in the Ridley–Jopling spectral classification that defines five spectral types of leprosy (TL, BT, BB, BL, and LL) [21]. At the tuberculoid pole, bacilli are scarce, whereas at the lepromatous pole, an inflammatory infiltrate containing Virchow's cells is replete with bacilli.

The specificity of skin biopsy specimens and histopathological examination ranges from 70% to 72%, but the sensitivity ranges from 49% to 70% [65, 66]. Among the 100 newly diagnosed untreated leprosy patients classified into the PB and MB groups according to the WHO classification, the sensitivity and specificity of the WHO classification were 63% and 85%, respectively, using the results of the slit-skin smear test and skin biopsy examination as gold standards [66]. This indicates that the accuracy of the present clinical classification can be further improved by adding more knowledge of diagnostic criteria.

The accuracy of skin biopsy examination depends on the appropriate selection of the location for biopsy, the representative sample size of skin biopsy, and the experience of the pathologist in leprosy examination [49, 63].

### 4.4. Lepromin Test

The lepromin test is an intradermal injection of the lepromin antigen (inactivated *M. leprae* extracted from lepromas) into the flexor surface of the forearm, and the delayed-type hypersensitivity (DTH) reaction is read at two time points. On inspection, there is an early (Fernandez) reaction and the other for a late (Mitsuda) reaction. Fernandez reaction was performed for 24 or 48 h. The Mitsuda reaction was read at 21 days and indicated resistance to *Bacillus*. A nodule measuring >5 mm indicates positivity [67, 68]. While patients with TT/BT evoke a strong DTH skin reaction, those with BL/LL fail to develop any skin reaction to lepromin [69]. A previous study showed that there was no difference in the mean reaction size between household contacts and noncontact testing with two soluble antigens of *M. leprae*, indicating that these antigens are not useful for the diagnosis of leprosy [69]. However, lepromin tests (lepromin H and lepromin A) are useful for confirming disease classification and prognosis [68]. Lepromin antigen tends to prime the immune response and is not specific for leprosy. Earlier skin test antigens for leprosy (lepromin A, Rees antigen, and Convit antigen) have been used for nearly 40 years and have been proven safe when used in humans [67]. Recently, two new skin test antigens, *Mycobacterium leprae* soluble antigens (MLSA) devoid of glycolipids, particularly lipoarabinomannan (LAM), called MLSA-LAM, and MLCwA (*M. leprae* cell wall-associated antigens), derived from *M. leprae* grown in armadillos, were produced. A clinical trial [68] showed that both antigens at low doses had a sensitivity of 20% and 25% in BT/TT leprosy patients, but specificity was 100% and 95%; at the high dose of both antigens, sensitivity was 10% and 15%, specificity was 70% and 60%, and BL/LL leprosy patients were anergic to the leprosy antigens [70].

Overall, early skin test antigens (lepromin A) for leprosy are safe when used in humans. Lepromin tests have poor accuracy for diagnosing leprosy in children. Lepromin tests have several shortcomings, including inconsistent readings due to soft rather than hard DTH reactions in some individuals, variation in potency between batches due to quality control issues, and lack of adequate sensitivity and specificity [68]. These tests are still useful for confirming classification and prognostic purposes.

### 4.5. PCR Tests

Active surveillance and early detection of the disease are imperative to prevent the spread of *M. leprae* and the burden of disability in society [12, 13]. Polymerase chain reaction (PCR) is a molecular technique used to detect deoxyribonucleic acid (DNA) in *M. leprae* and *M. lepromatosis*. A large proportion of early cases of leprosy in children remain AFB-negative on skin smear [71]. Such cases require additional techniques to confirm the diagnosis. In situ PCR on slit-skin smears is minimally invasive and less cumbersome than skin biopsy [72]. PCR is reported to have a higher sensitivity (87–100%) in patients with a positive BI or LL type; however, PCR sensitivity can be lower (30–83%) in patients with a negative BI or TT type [73].

Over the past 30 years, PCR methods have been developed to amplify various gene targets in *M. leprae*. PCR techniques have been used to detect possible environmental sources for the dissemination of *M. leprae* as well as the aerosol route of infection by means of nasal carriage [74–77]. The summary sensitivity of the PCR test was 75.3% (95% CI 67.9–81.5), and the specificity was 94.5% (91.4–96.5) [70].

Quantitative polymerase chain reaction (qPCR) is at least 20 times more sensitive than microscopic detection and has become increasingly important for early diagnosis and difficult-to-diagnose cases [78]. The summary sensitivity of the qPCR test was 78.5% (95% CI 67.9–89.2), and the specificity was 89.3% (61.4–97.8) [78, 79].

Thus, PCR is a molecular technique used to confirm the clinical diagnosis of leprosy. PCR is highly sensitive in patients with MB; however, its sensitivity is much lower in patients with PB. PCR is typically used to support the clinical diagnosis of leprosy. However, PCR is an expensive and laboratory-intensive technique, and most endemic countries cannot routinely perform it [74, 75].

### 4.6. Serology Test

Phenolic glycolipid 1 (PGL-1) is the most frequently studied antigen. The chemical structure of PGL-1 is a specific antigen of *M. leprae*. In 1981, serological tests using PGL-1 antigen for diagnosis were performed [80, 81]. This antigen in the cell wall is responsible for the immunological specificity of tests. PGL-1 serology, mainly using ELISA, rapid anti-PGL-1 assays, and lateral flow immunochromatographic assays, is considered surrogate marker for bacterial load and can aid in the clinical treatment of patients [81–84]. Anti-PGL-1 serology can identify patients early, provide early treatment, and reduce nerve damage and disability [84, 85]. PGL-1 antibody detection is useful in MB cases but is of little use in patients with PB. A previous study showed that the positive predictive value (PPV), negative predictive value (NPV), and sensitivity of the PGL-1 test in patients with MB were 43.4%, 94.6%, and 76.8% [86].

Overall, serological tests aim to detect PGL-1 antibodies that indicate *M. leprae* infections. These tests can be used to monitor the effectiveness of therapy, investigate the prevalence of diseases, and explore the distribution of infections in a population. Of all available serological tests, the summary sensitivity of ELISA was 63.8% (95% CI 55.0–71.8), and the specificity was 91.0% (95% CI 86.9–93.9); the summary sensitivity of the lateral flow test was 67.9% (95% CI 58.7–75.9); the summary sensitivity of the agglutination test was 72.8% (95% CI 55.8–83.7), and the specificity was 90.1% (95% CI 61.2–98.1) [70]. Although serological tests have not proven sufficient for diagnosing leprosy, several studies have been conducted in Cuba using kits for the detection of PGL-1 antibodies [62]. In the future, this tool may become a possible strategy for actively searching for new cases of leprosy in children.

### 4.7. Other Diagnostic Procedures

Electrophysiological nerve tests include nerve conduction studies and needle electromyography (EMG). These tests give us to provide information on the extent of nerve involvement and distribution of lesions [87]. The sensitivity of the nerve conduction test for leprosy diagnosis is 88%, whereas EMG used in conjunction with nerve conduction tests does not have a synergistic effect [88]. A nerve biopsy specimen examination is a confirmatory test in cases of pure neural leprosy and should be performed when leprosy is suspected and skin lesions are absent [89].

Ultrasonography of the peripheral nerves in leprosy to measure the extent of peripheral nerve thickening is a low-cost, noninvasive technology [90]. This test has been used for more than 20 years to diagnose leprosy. Ultrasonography of the nerves is a useful tool for objective assessment of nerve involvement in leprosy.

Taken together, clinical evaluation is the first step in the diagnosis of leprosy and is generally sufficient in most cases. The clinical diagnosis of leprosy is dependent on the recognition of disease signs and symptoms and is therefore only possible once the disease has manifested. Leprosy diagnosis in childhood may be more difficult than that in adults and involves confusing sensory testing. Untrained health practitioners may not be effective in recognizing the early signs of the disease. Therefore, obtaining travel and family history is important for the diagnosis of leprosy. The application of auxiliary laboratory-based tools is beneficial for supporting clinical diagnosis and classification.  Table 2 presents the sensitivity and specificity of different diagnostic tests.

**Figure 6:**
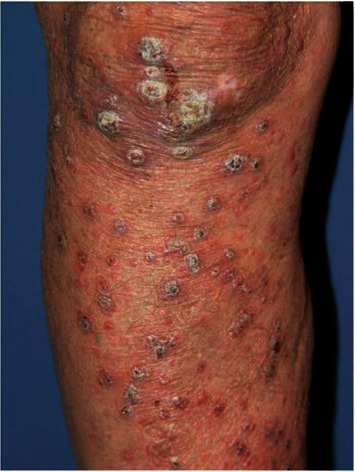
Lepromatous leprosy: skin lesions resembling guttate psoriasis, prurigo nodularis, or hypertrophic lichen planus (photograph courtesy of Kundakci, et al.) [91].

Because the disease presents diverse dermatologic and neurologic manifestations with a wide clinical spectrum, many such diseases should be considered in the differential diagnosis (Figure 6) [91]. The differential diagnoses of leprosy are broad and varied. Loss of pinprick or light-touch sensation is helpful in distinguishing leprosy from other disorders. Loss of sensation or neuropathy may not always be present, and obtaining a skin biopsy specimen can aid in differential diagnosis.

## 5. Treatment

A multidrug therapy regimen has been recommended by the WHO for the treatment of children according to age and the subdivision of these cases into paucibacillary and multibacillary forms (Table 3) [22]. Rifampicin, clofazimine, and dapsone (diaminodiphenyl sulfone) were used as the first-line treatments. Paucibacillary cases were treated for six months with rifampicin, dapsone, and clofazimine. Multibacillary cases were treated with rifampicin, dapsone, and clofazimine for 12 months. All patients received this drug combination monthly, under supervision.

In the United States, the regimens recommended by the National Hansen's Disease Program (NHDP) have a longer treatment period because of fewer cost restrictions and exclusion of clofazimine in PB treatment (Table 4) [92].

Minocycline, ofloxacin, and clarithromycin are among the drugs used as second-line treatments. The strengths of multidrug therapy include the prevention of resistance to dapsone, rapid decline in the infectivity of infected individuals, and low rate of recurrence and reactions [36]. Nonetheless, the treatment period is long and presents logistical problems, which makes adherence difficult to achieve.

Patients with leprosy and severe nerve damage, musculoskeletal disorders, and deformities may experience discrimination at school and difficulties in the social lives of patients with leprosy. Therefore, early diagnosis and treatment can reduce the transmission and disease sequelae in children. However, it is difficult for children to take medication in the form of tablets and capsules, and it is also impossible to chew capsules, which can subsequently lead to an inappropriate dose for treatment. The lack of medicines made for children in the form of oral solutions is a limiting factor for treatment adherence.

## 6. Prevention

### 6.1. Prophylactic Immunization

The aim of prophylactic immunization is to prevent infection, disease progression, or the administration of vaccines before or after exposure. Several vaccines, such as Bacille Calmette–Guérin (BCG), LepVax, and *Mycobacterium indicus pranii (MIP),* have proven effective [93]. However, currently, BCG is the only vaccine administered to prevent leprosy [94, 95]. In eastern India, a study was performed on patients with leprosy up to the age of 12 years attending a tertiary care hospital [96]. The nonvaccinated group had a significantly higher proportion of MB leprosy cases than the BCG-vaccinated group (*p*=0.0352). This study highlights the role of BCG vaccination in enhancing cell-mediated immunity (CMI). Overall, the protection of BCG vaccination against leprosy was estimated to range from 20% to 90% [97, 98].

However, leprosy remains prevalent in countries with widespread BCG vaccination programs, and as is the case for *tuberculosis* (TB), the protection afforded by BCG vaccination against leprosy appears to wane over time [99]. In addition, a study conducted from June 1987 to December 2006 to explore the effectiveness of BCG vaccination against leprosy showed that the protection of BCG vaccination appears to be better against the MB form than against the PB form [100]. However, the efficacy of the BCG vaccination remains controversial. Therefore, the development of more effective vaccines is essential. It can be used in addition to or instead of the BCG vaccine.

### 6.2. Chemoprophylaxis

In the 1960s, chemoprophylaxis using dapsone for leprosy exposure was reported [101]. For chemoprophylaxis, trials were performed with dapsone/acedapsone, rifampicin, and a combination of rifampicin, ofloxacin, and minocycline (ROM). Previous studies have indicated that a single dose of rifampicin (SDR) (25 mg/kg) administered to close contacts of new leprosy patients reduces the risk of developing clinical leprosy by 57% (95% CI 33–72) [102, 103]. Between 2015 and 2018, single-dose rifampicin postexposure prophylaxis (SDR-PEP) was conducted in the Union Territory of Dadra and Nagar Haveli (DNH) [104]. This study indicated that field-based leprosy research programs should focus on health systems.

In addition, another study conducted on results from Bangladesh who participated in this study showed that the additive protective effect of BCG and rifampicin was 80% (95% CI 50–92) [105]. This finding highlights the possibility that combined treatment strategies can reduce the incidence of leprosy. SDR postexposure prophylaxis was recommended by the WHO in 2018 and has been favored as postexposure prophylaxis for a few years; BCG vaccination may extend this [22]. However, the extent to which SDR suppresses excess leprosy cases after BCG vaccination is difficult to establish because many cases appear before SDR intervention [106, 107]. Further studies on chemoprophylaxis for leprosy prevention are needed [108].

## 7. Conclusion

Leprosy is a major public health concern worldwide. All healthcare workers must have basic knowledge of this disease to diagnose it, treat patients in a timely manner, and prevent disability and/or disease spread. The development of improved diagnostic and therapeutic methods for leprosy remains a significant challenge. This review provides some knowledge on the epidemiology, clinical diagnosis, and management of leprosy and makes it possible to eliminate leprosy worldwide. Further studies on the impact of leprosy on stigma, discrimination, and mental health are required.

## Figures and Tables

**Table 1:** Comparison of classifications of leprosy proposed by World Health Organization and Ridley–Jopling.

Classification		Brief description		
WHO (1987)	Ridley–Jopling [21]	Number of skin lesions	Neurological damage	Bacteriology: microscopic criteria
Paucibacillary				
1 skin lesion	Tuberculoid (TT)	Unique and infiltrated lesions	No neurological damage	Negative
2–5 skin lesions	Borderline tuberculoid (BT)	Stasis and hypopigmented lesions few or many lesions of varying size	Little neurological damage little or no neurological damage	Negative negative/1+
Multibacillary				
>5 lesions	Borderline borderline (BB)	Multiple lesions and maculopapular	Late thickening of the nerve	≧2+
	Borderline lepromatous (BL)	Multiple lesions, maculopapular, and nodules	Late thickening of the nerve	≧2+
	Lepromatous (LL)	Multiple lesions, maculopapular, and nodules	Late thickening of the nerve	≧2+

*Note.* 1+ or 2+: microscopic criteria when acid-fast bacilli were observed using Ziehl–Neelsen stain; negative, no acid-fast bacilli observed; BL, borderline lepromatous; BT, borderline tuberculoid; IND, indeterminate; LL, lepromatous leprosy; TT, tuberculoid leprosy.

**Table 2:** Comparisons between the sensitivity and specificity among the various diagnostic tests for leprosy.

Diagnostic tests	Sensitivity (%)	Specificity (%)
Slit skin smear test	50	100
Skin biopsy test	49–70	70–72
Lepromin test	20–25 (low dose) 10–15 (high dose)	95–100 (low dose) 60–70 (high dose)
PCR	67.9–81.5	91.4–96.5
Serological test (phenolic glycolipid 1)	55.0–71.8	86.9–93.9

**Table 3:** Summarized the treatment regimen recommended from World Health Organization.

Diagnosis	Population	Medication	Dose	Duration
Paucibacillary leprosy				
	Adults			
		Rifampicin	600 mg/month	6 months
		Clofazimine	300 mg/month + 50 mg/day	6 months
		Dapsone	100 mg/day	6 months
	Children (10–14 years)			
		Rifampicin	450 mg/month	6 months
		Clofazimine	150 mg/month + 50 mg/day	6 months
		Dapsone	50 mg/day	6 months
	Children (<10 years old or <40 kg)			
		Rifampicin	10 mg/kg/month	6 months
		Clofazimine	6 mg/kg/month + 1 mg/kg/day	6 months
		Dapsone	2 mg/kg/daily	6 months
Multibacillary leprosy				
	Adult			
		Rifampicin	600 mg/month	12 months
		Clofazimine	300 mg/month + 50 mg/day	12 months
		Dapsone	100 mg/day	12 months
	Children (10–14 years)			
		Rifampicin	450 mg/month	12 months
		Clofazimine	150 mg/month + 50 mg/day	12 months
		Dapsone	50 mg/day	12 months
	Children (<10 years old or <40 kg			
		Rifampicin	10 mg/kg/month	12 months
		Clofazimine	6 mg/kg/month + 1 mg/kg/day	12 months
		Dapsone	2 mg/kg/daily	12 months

**Table 4:** Summarization of the treatment regimen recommended by the National Hansen's Disease Program and the United States Health Resources and Services Administration.

Diagnosis	Population	Medication	Dose	Duration
Tuberculoid (TT and BT) (WHO classification paucibacillary)				
	Adults			
		Rifampicin	600 mg/day	12 months
		Dapsone	100 mg/day	12 months
	Children			
		Rifampicin	10–20 mg/kg/day (<600 mg)	12 months
		Dapsone	1 mg/kg/day	12 months
Lepromatous (LL, BL, and BB) (WHO classification multibacillary)				
	Adult			
		Rifampicin	600 mg/day	24 months
		Clofazimine	50 mg/day	24 months
		Dapsone	100 mg/day	24 months
	Children			
		Rifampicin	10–20 mg/kg/day (<600 mg)	24 months
		Clofazimine	1 mg/kg/day	24 months
		Dapsone	1 mg/kg/day	24 months

## Data Availability

Data sharing is not applicable to this article as no dataset was generated or analyzed during the current study.
